# The Watchful Mother

**Published:** 1876-05

**Authors:** 


### THE WATCHFUL MOTHER.

WE once sent a Sunday-school book by a lady patient of ours as a present to her little daughter. On inquiring afterwards how she liked it—"*Indeed, doctor, I did not give it to her, as I have not yet had time to read it myself.*" That mother soon passed away, and doubtless to the better land, and long years have passed away also, but we have never failed to admire that mother's heart as often as the remembrance of her ceaseless vigilance has occurred to us, accompanied with the earnest wish, that all parents should emulate that mother's care. Up to the age of fifteen at least, and as long after as affection for the parent will prevent the child from doing anything contrary to the known wishes of father or mother, no book should be read by a child without the parent's permission. Impressions are made for life, for eternity, on the mind, and heart, and memory of childhood- -impressions which mould the character for aye, or open up channels of thought which fix the destiny.

Untold mischief has been done to the minds and morals of the young by reading books on "Physiology" so-termed, causing apprehensions which have acted as a ceaseless torture to multitudes, until by consultation with honorable physicians, the groundless apprehensions have been removed, which had been excited by plausible falsities and brazen-faced untruths.

Equal care should be exercised as to the religious, moral, and miscellaneous reading of the young. Very few of our daily penny papers are fit to be read at the family fireside. Certainly not one in a dozen of all city weekly papers, not connected with a daily issue, but is chargeable justly with being made up with the veriest trash, to say nothing of their frequent obscenity, their slang, their spiteful hits at religion, its ministers, its professors, and the Bible itself.

A drop of water will ultimately wear through the solid rock, and drop by drop will empty the ocean, and so is the influence of the repeated exhibition of bits of sarcasm, and infidelity, and profanation, which portions of the press are steadily throwing out. Not only are the minds of the young injuriously affected by these things, but persons of maturity, of intellect, of mental culture, will suffer by them.

The death of *Percival* the poet, recalled to many memories his early promise, his later failure.—How, with a heart, a mind, a culture capable of achieving great things for humanity, his light went down in the night of misanthropy and almost atheism! What was it that froze the heart and made desolate the whole character of that gifted man? Reading in the spring-time of life, the obscenities of *Don Juan*, the malignant diatribes, the ranting atheism of Lord Byron. Had other books been placed in the hands of this unfortunate man at that critical period of his life—books which would have cherished the better feelings of his nature, which would have invited out his sympathies towards his brother man, he might have died a Howard or a Harlan Page, about whom sweet memories will arise for ages to come, instead of dying as he is said to have done, an uncomely oddity, a misanthrope, and an infidel.

PARENTS! *Have a ceaseless eye to what your younger children read.*

